# An exploratory analysis of 24-h movement behaviors in individuals with cancer completing a 12-week resistance exercise intervention

**DOI:** 10.1007/s00520-026-10637-7

**Published:** 2026-04-10

**Authors:** B. Nakfoor, H. Parker, J. H. Leach, C. M. Fairman

**Affiliations:** 1 University of Michigan Medical School, Ann Arbor, MI USA; 2 Department of Exercise Science, University of South Carolina, Columbia, SC USA; 3 Physical Activity for Treatment and Prevention Lab, College of Health and Human Sciences, Colorado State University, Fort Collins, CO USA

**Keywords:** Physical activity, Sleep, Sedentary behavior, Cancer, Exercise

## Abstract

**Purpose:**

Individuals with cancer often experience disrupted sleep, sedentary behavior, and reduced physical activity. This exploratory analysis examined the feasibility of continuous 24-h monitoring using wrist-worn accelerometers and characterized movement behaviors during a 12-week supervised resistance training program in individuals with cancer. We additionally aimed to evaluate whether daily movement behaviors (moderate-to-vigorous physical activity (MVPA), light physical activity (LPA), sedentary time, and sleep) differed between exercise and non-exercise days.

**Methods:**

Thirty individuals with cancer wore Axivity accelerometers continuously while participating in supervised resistance training (2–3 sessions/week). Feasibility was assessed via wear-time compliance. Movement behaviors were analyzed descriptively across exercise and non-exercise days throughout the intervention.

**Results:**

Participants demonstrated high adherence to continuous monitoring, with valid wear data on 70% of all days of the intervention. Within-person comparisons revealed significantly higher MVPA (+3.3 min) and LPA (+10.9 min) on exercise days. No significant changes were observed in sleep duration or sedentary time across the intervention or between exercise and non-exercise days.

**Conclusions:**

Continuous wrist-worn accelerometry is a feasible method for long-term behavioral monitoring in individuals with cancer. Supervised resistance training produced modest acute increases in physical activity but did not impact sleep or sedentary time.

## Introduction

It is estimated that there are over 18 million individuals currently living with a history of cancer in the United States alone, a figure projected to continuously expand in the coming years [1, 2]. With an increasing number of individuals living with the late and long-term effects of cancer and its treatment [3–9] (e.g., persistent fatigue [10–16], physical deconditioning [4, 17–23], disrupted sleep, and reduced quality of life), there is a critical need for supportive care strategies [5, 24–28].

One such evidence-based supportive care strategy includes structured, leisure time physical activity (i.e., exercise). Numerous professional bodies have issued guidelines that endorse exercise in cancer care to mitigate treatment-related side effects and support long-term recovery [29–32]. The efficacy of exercise interventions for improving various physical and psychosocial outcomes is well established [33] and exercise programming for cancer survivors is widely available [34]. However, there exists little information regarding how exercise training may impact cancer survivors’ behaviors day to day. This full spectrum of behaviors (i.e., the 24-h movement paradigm) which includes exercise and non-exercise physical activity, sedentary behavior, and sleep may have important implications for cancer survivors in terms of key outcomes such as fatigue [35].

Previous weight loss literature has postulated that exercise may result in behavioral compensation, in which individuals do less non-exercise physical activity in response to exercise training sessions [36]. Sleep and circadian literature suggest that there may be a positive effect of exercise on sleep [37]. Despite these previously established relationships in other populations, given the high prevalence of cancer-related symptoms and side effects such as fatigue, there may be a differential effect of exercise training on daily activity behaviors among cancer survivors. Previous observational studies in cancer populations have produced mixed findings regarding the benefits of reallocating time spent in moderate-to-vigorous physical activity (MVPA) for outcomes such as fatigue and quality of life [38, 39] and some evidence supports beneficial effects on body mass index [40, 41]. Although these studies provide early promise that 24-h movement behaviors may influence key health outcomes in cancer survivors, to our knowledge, there is a scarcity of studies that have examined the effect of exercise training on day-level 24-h activity behaviors among cancer survivors.

Previous studies using accelerometry to characterize 24-h movement behaviors among cancer survivors have typically been done in observational settings or at discrete timepoints before and after interventions, largely for 7 days at the beginning and at the end of interventions [35, 38–41]. While informative, this approach overlooks daily fluctuations in behaviors and provides limited insight into how exercise sessions during an intervention impact non-exercise physical activity, sedentary time, and sleep [35]. Thus, there is a need to examine how exercise may affect 24-h movement behaviors throughout the course of an exercise intervention for cancer survivors, rather than solely at the beginning and at the end [35]. Such an approach would allow for the examination of real-time behavioral adaptations, intra-individual variability, and differences between exercise and non-exercise days, offering a more nuanced understanding of the interaction between physical activity, sleep, and sedentary behavior in response to structured exercise. To address the gaps outlined above, the primary aim of this study was to compare 24-h movement behaviors, (i.e. sedentary time, sleep, and physical activity), on days participants attended supervised exercise sessions versus non-session days during a 12-week intervention in individuals with cancer.

## Methods/design

**Fig. 1:**
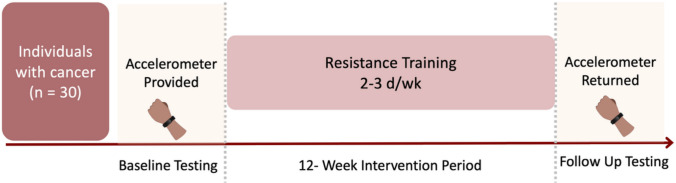
Overview of study design

This is a sub-analysis of an ongoing randomized controlled trial investigating the effects of resistance exercise on physiological and psychosocial wellbeing in individuals treated for cancer. In this single-group, exploratory analysis, we examined 24-h movement behavior over a 12-week supervised resistance exercise program in a subset of 30 participants (Fig. 1). Movement behavior and sleep quality were continuously assessed throughout the intervention using wrist-worn accelerometers. Assessments were initiated at baseline and maintained through week 12 to evaluate both behavioral trends and device adherence. The study protocol was approved by the Advarra Institutional Review Board (Protocol #Pro00079265), and all participants provided written informed consent prior to the initiation of any study procedures.

### Participant recruitment, screening and eligibility

Participants were recruited from a private radiation oncology practice located in Southwest Florida. Recruitment occurred between June and November 2024, using a combination of physician referrals, printed flyers displayed throughout the facility, and word-of-mouth from healthcare providers. Recruitment materials (e.g., flyers, brochures) were placed in high-traffic areas of the radiation oncology clinic, including waiting rooms, check-in desks, and infusion bays. Individuals expressing interest in the study were contacted by study staff to discuss the study objectives, potential risks and benefits, and to determine eligibility.

Eligible participants included adults (≥ 18 years old) of any sex, who were currently undergoing or had completed primary cancer treatment within the past year (including chemotherapy, radiation therapy, surgery, immunotherapy, and/or targeted therapy). Exclusion criteria were: (1) unwillingness to attend in-person exercise sessions at the on-site wellness center; (2) presence of neuromuscular, cardiovascular, or psychological conditions that would contraindicate exercise participation based on the American College of Sports Medicine’s (ACSM) guidelines for pre-exercise screening [42]; and (3) inability to read or comprehend English. No additional exclusion criteria were applied based on concurrent treatment for non-cancer-related medical conditions.

### Intervention

The resistance exercise intervention was delivered by trained study staff with prior experience in implementing exercise programs for individuals with cancer. Exercise sessions were conducted at a dedicated exercise oncology facility affiliated with a radiation oncology practice in Southwest Florida. Facility access was available from 8:00 AM to 5:00 PM, Monday through Friday, and from 8:00 AM to 1:00 PM on Saturdays to accommodate participant schedules. Each participant completed a 12-week supervised resistance training program, consisting of two to three exercise sessions per week on non-consecutive days. Each session lasted approximately 45 min and was delivered in a small-group format, maintaining an instructor-to-participant ratio of approximately 1:3–5.

The intervention targeted fundamental movement patterns, including upper-body pushing and pulling in both the vertical and horizontal planes, lower-body hinging and squatting, and core strengthening movements [42]. Exercises were performed using a combination of free weights such as dumbbells, kettlebells, and barbells, bodyweight movements, and exercise machines as needed to accommodate individual limitations (Table 1). This approach has been used successfully in ours and others’ prior work in exercise oncology [42–51]. Exercise selection and program modifications were individualized based on each participant’s movement competency, pre-existing impairments, and clinical presentation. Study staff assessed participants’ performance and symptoms throughout each session and adjusted exercises as necessary to ensure safety and appropriateness. Load prescription was based on the self-determined repetitions in reserve (SD RIR) [42, 43]. Participants were introduced to the SD RIR concept during the initial sessions through structured instruction and anchoring exercises designed to calibrate their perceived exertion. Participants were encouraged to select a load that allowed them to complete the prescribed number of repetitions while leaving a specific number of repetitions in reserve, consistent with the session’s goals.

**Table 1:** Overview of exercise selection template

	Variation 1	Variation 2	Variation 3	Variation 4	Variation 5
Hip Hinge	BW Hinge	DB RDL	Trapbar Deadlift	Barbell Deadlift	
Squat	BW Chair Squat	Weighted Chair squat	BW Squat	Goblet Squat	
Single Leg	Supported Single Leg Balance	Unsupported Single Leg Balance	Supported Split Squat	BW Split Squat	Weighted Split Squat
Horizontal Push	High Box pushup	Low Box pushup	BW Pushup	DB Bench Press	
Horizontal Pull	Seated Horizontal Band Pull	Chair Supported DB Row	TRX Row	Chest-Supported Row	Barbell Bent Over Row
Vertical Push	Front Raises	Seated DB Shoulder Press	Standing DB Shoulder Press	Half-Kneeling DB Shoulder Press	
Vertical Pull	Seated Vertical Band Pull	Lat Pulldown	Assisted Pullup	Unassisted Pullup	
Core	Pallof Press	Deadbugs	Weighted Side Bends	Planks	Renegade Rows

*BW* Bodyweight, *DB* Dumbbell, *RDL* Romanian Deadlift

Progression and regression of exercises were guided by participant performance and clinical judgment of staff members. When participants consistently completed the prescribed number of repetitions at a given load over two or more sessions, the load was incrementally increased (typically by 2–10%) while maintaining the target repetition range (Table 2) [42–45]. Conversely, if participants exhibited signs of excessive fatigue, symptom exacerbation, or other adverse effects, the load was reduced, or the exercise was modified to allow safe completion within the target repetition range. Outside of the supervised sessions, participants were encouraged to remain physically active according to their comfort levels through activities such as walking or cycling; however, no formal unsupervised aerobic or resistance training program was prescribed outside of the supervised sessions.

**Table 2:** Progression of volume/load across intervention

Week	Sets	Reps	Loading (SD RIR)
1	2	12	~ 3–4
2–4	3	12	~ 2
5–8	3	10	~ 2
9–12	3	8	~ 2

### Outcomes

All data were collected by trained study staff at the exercise oncology facility. Baseline assessments included demographic information and select cancer-related and medical characteristics, which were obtained through self-report and verified via medical records when available. Anthropometric measurements were conducted at baseline using a calibrated stadiometer to assess height and a calibrated scale for body mass.

#### 24-h movement behaviors

Participants wore an Axivity AX3 triaxial accelerometer for 24 h per day for the duration of the 12-week intervention to measure physical activity, sleep, and sedentary behavior (Axivity Ltd., Newcastle, UK). Devices were initialized to collect raw acceleration data at a sampling frequency of 25 Hz and configured to record continuously over the entire 12-week intervention period. Participants were instructed to wear the device on their non-dominant wrist, secured with a hypoallergenic, adjustable strap, and to wear it continuously, including during sleep and routine daily activities. Devices were water-resistant and could be worn during showering; participants were instructed to remove the device only during prolonged submersion in water (e.g., swimming or bathing). Wrist-wear was selected based on participant convenience, expected compliance, and compatibility with validated algorithms for estimating 24-h movement behaviors. During orientation, study staff emphasized the importance of consistent wear and provided both verbal and written instructions on proper device use. Participants were asked to return the device only if issues arose and were encouraged to contact study staff with any concerns.

Data were processed with GGIR (version 3.2.0) in R (version 4.3.1). We used GGIR variables from the part 5-day summary and extracted the variables as follows: ‘dur_spt_sleep_min’ was used for sleep duration, ‘dur_day_total_LIG_min’ was used for light physical activity (LPA), and ‘dur_day_total_MOD_min’ + ‘dur_day_total_VIG_min’ was used for moderate-to-vigorous physical activity. We used the part 5-day summary because it includes information for sleep and physical activity at the day-level rather than the person-level. A valid day of accelerometry was defined as ≥ 10 h per day of wear. Intensity thresholds from Hildebrand were used to classify light, moderate, and vigorous physical activity [52–54]. We considered moderate physical activity or more to be moderate-to-vigorous physical activity. The HDCZA algorithm was used to detect sleep periods. A valid night of sleep duration had to be > 3 h.

### Statistical analysis

All statistical analyses were performed using STATA software (version 16.1). No formal power calculation was conducted for this exploratory analysis. Rather, the sample size was determined through a combination of logistical, time, and cost constraints. Descriptive statistics were used to summarize participant demographic and medical characteristics. Continuous variables were described using means and standard deviations (SD), and categorical variables were summarized using frequencies and percentages. Two-level multilevel linear regression models were used to predict each outcome (i.e., minutes of MVPA, LPA, SB, and sleep duration) from a binary intervention attendance day-level variable (attended vs. did not attend that day). Intervention attendance was disaggregated using person-mean centering to represent between- and within-person effects. For this study, to examine how an exercise session affected 24-h movement behaviors, we focused on interpreting the within-person variable to represent days that a participant attended the intervention versus days that they did not attend the intervention while controlling for the between-person effect that represented the proportion of days a participant attended the exercise intervention. The within-person variable estimated the change in the outcome (i.e., minutes of MVPA, LPA, SB, and sleep duration) on days when a participant attended the intervention compared to days that they did not attend the intervention. Models accounted for the nested data with days (level 1) nested within participants (level 2) by including a random effect for participant. Separate models were conducted for each dependent variable of MVPA, LPA, SB, and sleep duration.

## Results

A total of 30 individuals with cancer were included in this analysis. The sample included a range of tumor types, with breast and prostate cancers each representing the most common diagnosis (n = 10, 33.3% for each). The mean age of participants was 66.2 years (SD = 9.8), and there were an equal number of males and females (*n* = 15). The average body mass index (BMI) was 26 kg/m^2^ (SD = 5.9). Additional baseline demographic and clinical characteristics are presented in Table 3.

**Table 3:** Participant sample characteristics

Characteristics	Total (*N* = 30) *n* (%) or m ± sd
**Biological Sex**	
Male	15 (50%)
Female	15 (50%)
**Age**	66.2 ± 9.8
**BMI**	26 ± 5.9
**Ethnicity**	
White, not of Hispanic origin	27 (90%)
Hispanic	3 (10%)
**Cancer Type**	
Breast	10 (33.3%)
Prostate	10 (33.3%)
Lymphoma	2 (6.7%)
Head and Neck	2 (6.7%)
Gastrointestinal	2 (6.7%)
Other	4 (13.3%)
**Cancer Stage**	
I	10 (33.3%)
II	8 (26.7%)
III	4 (13.3%)
IV	5 (16.7%)
X	2 (6.7%)
IE	1 (3.3%)
**Time since Diagnosis (months)**	4.4 ± 2.6
**Active Treatment (Y/N)**	17 (56.7%)
Chemotherapy	3 (10%)
Radiotherapy	14 (46.7%)
Hormone Therapy	12 (40%)
Immunotherapy	1 (3.3%)
Targeted Therapy	1 (3.3%)
**Past Treatment**	
Surgery	14 (46.7%)
Previous Chemotherapy	8 (26.7%)
Previous Radiation	9 (30%)
Previous Hormone Therapy	2 (6.7%)

Participants had a mean attendance rate of 76.4% (SD = 14.9) and a relative dose intensity of 84.7% (SD = 17.3) across the intervention (Table 4). The most common reasons for missed sessions included personal reasons (4.2%), conflicting appointments (1.6%), and vacation (1.5%). Modifications to the prescribed exercise dose were infrequent, but most commonly involved changes to exercise selection (8.1%) and removal (0.3%). Reasons for dose modification were due to lower extremity pain (3.5%) or unspecified (4.7%).

**Table 4:** Attendance rate and relative dose intensity of participants throughout the intervention

	**Mean (SD)**			
**Attendance**	76.4 (14.9)			
**Relative Dose Intensity**	84.7 (17.3)			
**Missed Sessions**	**No. Participants**	**Percentage**	**No. Sessions**	**Percentage**
Vacation	5	16.7	15	1.5
Fatigue	1	3.3	1	0.1
Injury	1	3.3	3	0.3
Nausea	2	6.7	12	1.2
General Illness	3	10	12	1.2
Conflicting Appointments	5	16.7	16	1.59
General Pain	1	3.3	1	0.1
Other	19	63.3	43	4.3
Not Recorded	23	76.7	93	9.23
Personal	6	20	42	4.2
**Dose Modification**				
Sets	1	3.3	2	0.2
Reps	1	3.3	1	0.1
Weight	5	16.7	9	0.9
Exercise selection	19	63.3	82	8.1
Exercise removal	3	10	3	0.3
**Modification Reason**				
Fatigue	1	3.3	1	0.1
Nausea	1	3.3	1	0.1
Upper extremity pain	2	6.7	5	0.5
Lower extremity pain	12	40	35	3.5
Other	16	53.3	47	4.7
Not Recorded	10	33.3	13	1.3

**Fig. 2:**
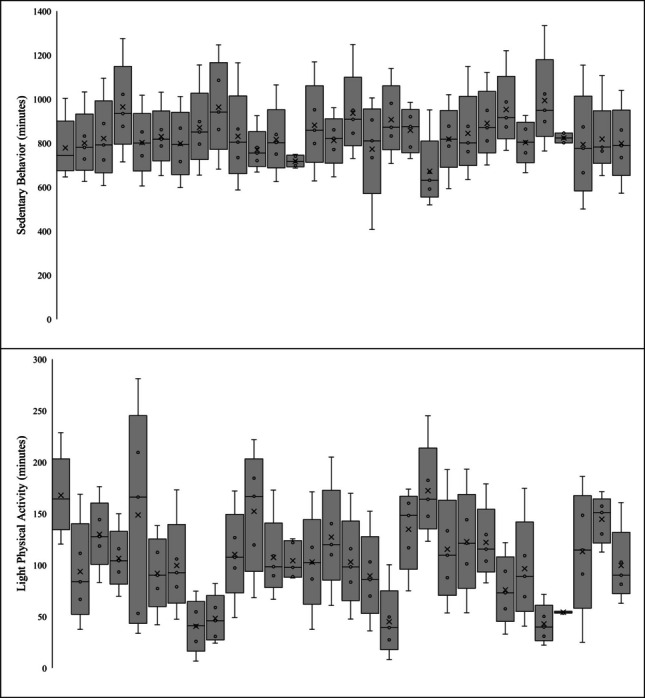

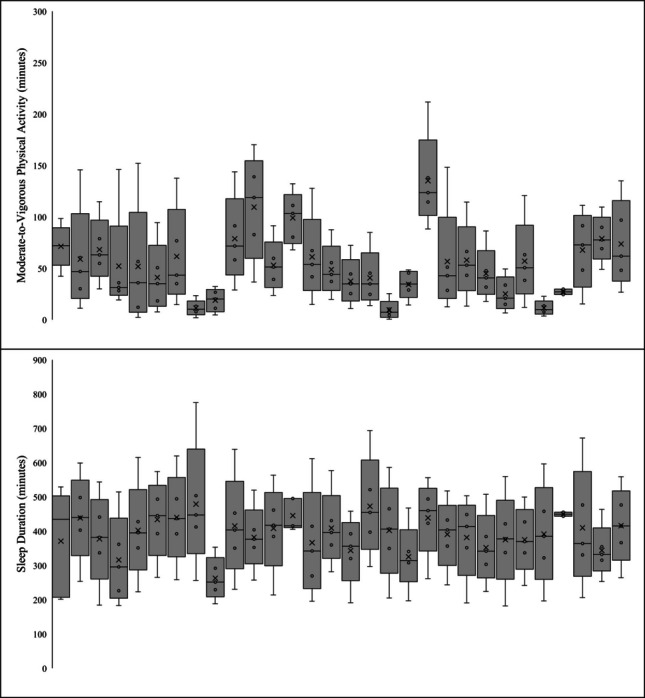
Within-person variability of movement behaviors across the intervention

**Fig. 3:**
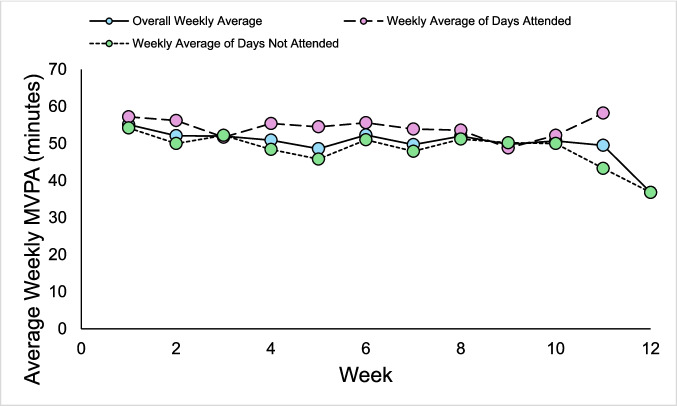
Weekly averages of MVPA across the intervention

Descriptive data, including means and SDs, of participants 24-h movement behaviors are presented in Table 5. Data included 1,715 valid days of accelerometry. Wear-time compliance was 70.2%, with participants contributing an average of 57/84 days (range = 1–72). On average, participants attended the intervention of n = 19 days (range = 0–31). Overall, participants had a daily average of 51.1 ± 34.4 min of MVPA, 104.8 ± 45.1 min of LPA, 829.3 ± 117.8 min of SB, and 398.4 ± 85.9 min of sleep duration (6.6 ± 1.4 h) per day/night. More specifically, on days when participants attended the intervention, they had a daily average of 54.2 ± 34.1 min of MVPA, 115.7 ± 44.7 min of LPA, 829.5 ± 113.9 min of SB, and 394.8 ± 81.5 min of sleep duration (6.6 ± 1.4 h) per day/night. Contrastingly, on days when participants did not attend the intervention, they had a daily average of 49.8 ± 34.5 min of MVPA, 99.6 ± 44.4 min of LPA, 829.2 ± 119.7 min of SB, and 400.1 ± 88.0 min of sleep duration (6.7 ± 1.5 h) per day/night. Variability, including minimum, IQR, and maximum values, in 24-h movement behaviors across the 12-week intervention per participant is presented in Fig. 2. Weekly averages, including overall, only days the intervention was attended, and only days the intervention as not attended, in 24-h movement behaviors across the 12-week intervention are presented in Fig. 3.

**Table 5:** Descriptives of 24-h movement behaviors (*N* = 30 participants; *n* = 1729 days)

Movement Behavior	Mean	SD	Median	Min	Max
Overall					
LPA	104.8	45.1	102.3	6.8	281.2
MVPA	51.2	34.4	45.5	0.5	211.7
Sedentary Behavior	829.3	117.8	821.5	407.4	1334.0
Sleep Duration	398.4	86.0	402.7	181.9	775.4
Did Not Attend Intervention Days					
LPA	99.6	44.4	99.3	6.8	277.7
MVPA	49.8	34.5	44.4	0.5	211.7
Sedentary Behavior	829.2	119.7	823.3	407.4	1248.6
Sleep Duration	400.1	88.0	405.5	181.9	775.4
Did Attend Intervention Days					
LPA	115.7	44.7	107.8	18.8	281.2
MVPA	54.2	34.1	46.6	6.3	170.3
Sedentary Behavior	829.5	113.9	815.7	500.5	1334.0
Sleep Duration	394.8	81.5	396.7	185.3	685.7

### Mixed effects model for intervention attendance on 24-h movement behaviors

Mixed effects model output for all models are presented in Table 6. These models evaluated within-person differences in daily 24-h movement behaviors as a function of exercise intervention attendance, while accounting for the nested data structure (days nested within individuals). After adjusting for between-person variation, within-person effects revealed that on days participants attended the intervention, they engaged in significantly more physical activity compared to days they did not attend. Specifically, participants accrued an additional 3.3 min of moderate-to-vigorous physical activity (MVPA) (SE = 1.1, p = 0.002, 95% CI: 1.2 to 5.4), and 10.9 additional minutes of light physical activity (LPA) (SE = 1.5, p = 0.000, 95% CI: 7.9 to 13.9) on intervention days.

**Table 6:** Effects of intervention attendance on 24-h movement behaviors

	Coef	Std. Error	*p*	95%CI	
**LPA**					
Intervention Attendance	10.9	1.5	0.000	7.9	13.9
Proportion of Days Intervention Attended*	64.7	41.5	0.119	−16.6	146.0
Age	−0.8	0.6	0.218	−2.0	0.5
BMI	−1.2	1.0	0.195	−3.1	0.6
Treatment Status	1.5	11.0	0.889	−20.1	23.2
Cancer Type	−2.2	1.8	0.238	−5.8	1.4
Intercept	171.9	56.5	0.002	61.3	282.6
**MVPA**					
Intervention Attendance	3.3	1.1	0.002	1.2	5.4
Proportion of Days Intervention Attended*	4.6	34.3	0.894	−62.6	71.8
Age	−1.4	0.5	0.008	−2.4	−0.4
BMI	−0.7	0.8	0.369	−2.3	0.9
Treatment Status	−7.0	9.3	0.452	−25.1	11.2
Cancer Type	−0.5	1.6	0.736	−3.6	2.5
Intercept	165.3	47.0	0.000	73.1	257.5
**Sedentary Behavior**					
Intervention Attendance	2.5	5.0	0.617	−7.4	12.4
Proportion of Days Intervention Attended*	7.9	94.5	0.934	−177.3	193.1
Age	3.7	1.4	0.007	1.0	6.4
BMI	3.0	2.1	0.146	−1.1	7.1
Treatment Status	5.4	23.8	0.822	−41.3	52.1
Cancer Type	0.2	4.0	0.96	−7.6	8.0
Intercept	496.6	124.8	0.000	252.0	741.3
**Sleep Duration**					
Intervention Attendance	−2.4	4.0	0.555	−10.3	5.5
Proportion of Days Intervention Attended*	−98.5	66.0	0.136	−227.8	30.8
Age	−1.4	0.9	0.125	−3.3	0.4
BMI	−2.5	1.4	0.079	−5.3	0.3
Treatment Status	2.3	16.2	0.885	−29.4	34.1
Cancer Type	1.1	2.7	0.690	−4.2	6.4
Intercept	580.3	85.8	0.000	412.2	748.5

^*^Proportion of days attended allows the model to control for between-person effects in order to isolate the day-level within-person effect

No significant within-person differences were observed for sedentary behavior or sleep duration on exercise vs. non-exercise days. On average, sedentary time did not significantly decrease (coef. = 2.5 min, SE = 5.0, p = 0.617, 95% CI: −7.4 to 12.4), nor did sleep duration significantly vary (coef. = –2.4 min, SE = 4.0, p = 0.555, 95% CI: −10.3 to 5.5) based on intervention attendance.

## Discussion

The primary aim of this exploratory analysis was to compare 24-h movement behaviors on days participants attended supervised exercise sessions versus non-session days during a 12-week intervention in individuals with cancer. The results of our within-person analyses revealed significantly greater engagement in MVPA and LPA on days participants attended supervised exercise sessions. Specifically, participants accumulated an average of 3.3 additional minutes of MVPA and 10.9 more minutes of LPA on exercise days compared to non-exercise days. These changes, while modest in absolute terms, may represent a meaningful shift in energy expenditure or functional capacity, particularly for individuals experiencing cancer-related fatigue or physical deconditioning [55]. Prior work suggests even small increases in daily MVPA and LPA are associated with improved physical function and quality of life in individuals with chronic conditions [55–62]. However, consistent with other literature [63–68], whilst supervised exercise sessions produce reliable increases in daily activity, these increases do not extend to non-exercise days, highlighting the need for additional behavioral strategies to promote habitual activity. Future interventions might incorporate behavioral techniques (e.g., goal-setting, prompts, or home-based assignments), along with barrier problem-solving and strategies to enhance self-efficacy (e.g., mastery experiences, reinforcement, peer modeling), to encourage more independent activity outside of supervised sessions, thereby converting the acute boosts into a more comprehensive lifestyle change [69–76].

Despite increases in physical activity, no significant differences were observed in sedentary behavior or sleep duration between exercise and non-exercise days. Our findings suggest that while structured exercise is effective at increasing activity levels, it may not be sufficient on its own to modify other domains of the 24-h movement profile. These findings are consistent with prior literature reporting inconsistent effects of exercise-only interventions on sleep and sedentary behavior in cancer survivors [27, 28, 77–84]. These findings suggest that while exercise interventions may increase activity levels, additional components are likely needed to influence sedentary time and sleep. Given that prolonged sedentary time and poor sleep quality have been independently associated with poorer health outcomes in cancer survivors, including increased mortality risk and reduced physical function [38, 78, 85, 86], addressing sedentary behavior explicitly in future interventions may be a critical target for improving survivorship health trajectories. Multi-component interventions that include coaching, self-monitoring, or behaviorally informed support may be better to target behaviors such as prolonged sitting or disrupted sleep [35, 70–79, 81, 84, 87–89].

Although we only examined physical activity, sedentary time, and sleep, our preliminary findings suggest that continuous wrist-worn activity monitoring is practical and may offer value for tracking real-time behavioral adaptations during exercise interventions in oncology. Wearable accelerometry provides objective 24-h movement profiles that could support more personalized exercise guidance. While symptoms were not assessed in this study, future work should integrate patient-reported outcomes to explore how fluctuations in physical activity relate to fatigue, pain, or mood, or acute exercise response [35, 90–93]. This integration could help determine whether movement data can serve as a proxy for symptom monitoring and support adaptive care interventions in this population [94–97].

Standard practice for evaluating 24-h movement behaviors is to collect data on each behavior across multiple days (commonly 3–4 days) and aggregate these values into a single metric for each behavior. Whilst this approach offers interesting insights, it obscures day-to-day variability and prevents exploration of how daily factors (e.g. intervention attendance, fatigue, symptom burden, stress, etc.) may influence behavior. Further, evaluation of these behaviors typically occurs at baseline and post-intervention, rather than continuously monitored throughout, potentially missing out on insights in variability in behaviors. In contrast, participants in our study were asked to wear the accelerometer for the entirety of the intervention duration, achieving a mean of 71.4% valid wear days (60 out of 84; range = 2–73), demonstrating high adherence relative to prior studies that have largely relied on short-term (e.g., 7-day) monitoring protocols [90, 98, 99]. These findings support the feasibility of sustained accelerometry use in older adults treated for cancer [35, 38–41] and highlight its potential as a practical method for capturing nuanced, real-time behavioral data that can inform more personalized interventions [99–103]. As cancer care increasingly integrates remote monitoring and tailored exercise prescriptions, continuous accelerometry may enhance survivorship care by providing ongoing insight into daily movement behavior during lifestyle interventions (rather than anchored before and after an intervention period). Of note, most accelerometers in this intervention lost charge in week 11; therefore, future studies may consider adding a mid-intervention charge or alternatively, a mid-intervention reassignment of fully charged accelerometers to ensure that data is able to be collected throughout the entirety of the intervention.

## Limitations

This study has several limitations worth acknowledging. First, the single-group, non-randomized design precludes causal inference, as we cannot definitively attribute observed changes to the intervention without a control group. Second, the exclusive focus on resistance training limits generalizability to other exercise modalities, such as aerobic or mixed-mode programs. Third, participants self-selected into an exercise trial, likely resulting in a sample that was more motivated, health-conscious, and physically capable than the general survivor population. This selection bias, commonly observed in exercise oncology studies [69], limits the applicability of our findings to broader populations. Future studies should employ randomized controlled designs with more diverse and representative samples to validate and extend these preliminary results.

An important consideration in the interpretation of our findings is the ability of accelerometry to accurately capture resistance training activity. Although sessions in this study lasted 45 to 60 min, including warm- and cool-down our data showed only modest increases in MVPA (approximately 4 min) and LPA (approximately 14 min) on exercise days. Resistance exercise primarily involves acute bouts (i.e., sets of multiple repetitions) of exercise that are typically done in static positions. As such, accelerometers may not fully capture load lifted, sets, repetitions, or effort level, all critical components of resistance training stimulus and subsequent adaptations [104]. Accelerometers primarily measure locomotor activity and do not inform body posture (i.e., performing exercises when seated or standing still) [105]. Consequently, using accelerometry alone may not fully characterize the intensity or quantity of resistance training performed [106, 107]. This has important implications for interpreting the findings of this study, as the modest changes in physical activity detected by accelerometry likely underestimate the actual training volume and intensity completed. Nonetheless, capturing movement behavior at the day level remains valuable for understanding broader patterns in 24-h movement behaviors and the potential impact of participating in a structured exercise intervention, particularly on additional behaviors such as sedentary behavior and sleep.

## Conclusion

This exploratory analysis demonstrates that continuous wrist-worn accelerometry is a feasible and informative tool for monitoring daily movement behaviors during a 12-week resistance exercise intervention in individuals with cancer. While supervised sessions led to meaningful increases in physical activity on exercise days, these effects did not extend to non-exercise days or other movement domains such as sedentary behavior and sleep. These findings underscore the potential of integrating behavioral support strategies to promote broader and more sustained lifestyle changes. As oncology care continues to evolve toward remote and personalized models, the inclusion of continuous monitoring and targeted behavioral interventions may offer a promising avenue to optimize exercise adherence, daily movement, and overall survivorship outcomes.

## Data Availability

No datasets were generated or analysed during the current study.
